# Arbitrarily tight $\alpha$BB underestimators of general non-linear functions over sub-optimal domains

**DOI:** 10.1007/s10898-018-0632-3

**Published:** 2018-03-29

**Authors:** N. Kazazakis, C. S. Adjiman

**Affiliations:** Department of Chemical Engineering, Centre for Process Systems Engineering, Imperial College London, London, SW7 2AZ UK

**Keywords:** $\alpha$BB, Subenergy, Underestimator, Eigenvalue

## Abstract

In this paper we explore the construction of arbitrarily tight $\alpha$BB relaxations of $C^2$ general non-linear non-convex functions. We illustrate the theoretical challenges of building such relaxations by deriving conditions under which it is possible for an $\alpha$BB underestimator to provide exact bounds. We subsequently propose a methodology to build $\alpha$BB underestimators which may be arbitrarily tight (i.e., the maximum separation distance between the original function and its underestimator is arbitrarily close to 0) in some domains that do not include the global solution (defined in the text as “sub-optimal”), assuming exact eigenvalue calculations are possible. This is achieved using a transformation of the original function into a $\mu$-*subenergy* function and the derivation of $\alpha$BB underestimators for the new function. We prove that this transformation results in a number of desirable bounding properties in certain domains. These theoretical results are validated in computational test cases where approximations of the tightest possible $\mu$-subenergy underestimators, derived using sampling, are compared to similarly derived approximations of the tightest possible classical $\alpha$BB underestimators. Our tests show that $\mu$-subenergy underestimators produce much tighter bounds, and succeed in fathoming nodes which are impossible to fathom using classical $\alpha$BB.

## Introduction

In this paper we discuss the problem of locating a global minimum of a general $C^2$ non-linear function $f:X\rightarrow F\subset \mathbb {R},\,X=\{\varvec{x}:\varvec{x}\in [\varvec{x}^L,\varvec{x}^U]\}\subset \mathbb {R}^N$, denoted as problem *P*:

1 $$\begin{aligned} P~:~~\underset{\varvec{x}\in X}{\min } f(\varvec{x})\end{aligned}$$

Looking past its simple description, problem *P* is generally very difficult to solve to global optimality in a deterministic way (it is, in fact, NP-hard [55]), even for problems of low dimensionality. Over the last fifty years, considerable effort has been invested towards inventing tools to solve this problem, resulting in a multitude of methods (e.g., [19, 26, 34, 39, 40]). Although modern deterministic global optimization methods guarantee that the global optimum of many general $C^2$ optimization problems can always be located with certainty, and in finite time, this time may be too long to be viable in practice. Even if an algorithm suitable for handling a particular mathematical structure exists, the required solution time is often prohibitively expensive for many practical problems.

The prevalent family of methods, amidst the rich thesaurus of proposed algorithmic procedures, is that of branch-and-bound algorithms [26, 29], the building blocks of which have seen rapid development over the last few decades (e.g., [6, 7, 11, 15, 18, 24, 30, 31, 37, 38, 45, 50, 51, 54, 59, 60]). The central concept behind this set of methods is a divide-and-conquer approach, in which the solution space is sequentially subdivided and bounds on the new sub-domains are derived. This information is then used to fathom sub-regions of the solution space based on specific criteria. This process is repeated iteratively until a convergence criterion is satisfied.

Problem *P* may be solved using branch-and-bound if a *convex relaxation* [52] of *f* is generated over *X*. This may be accomplished by introducing additional variables and constraints to the relaxed problem, or by generating a function $\breve{f}$ giving the relaxed problem:

2 $$\begin{aligned} \breve{P}~:~~\underset{\varvec{x}\in X}{\min } \breve{f}{(\varvec{x})} \end{aligned}$$

This latter approach is adopted in this paper. The new function $\breve{f}:X\rightarrow \breve{F}$ must possess the following properties: (1) it is convex on *X*, (2) $\breve{f}(\varvec{x})\le f(\varvec{x}),~\forall \varvec{x}\in X$, and (3) the value at the global minimum of the convex function $\breve{f}{(\varvec{x})}$, which is a valid lower bound on the value of $f(\varvec{x})$, is guaranteed to improve as *X* becomes smaller. Convexification of non-convex non-linear functions is a technique commonly used to derive bounds in the context of branch-and-bound because solving the convex problem $\breve{P}$ with a local solver returns a deterministic *lower bound* on the value of *f* in the domain of interest.

Functions may be convexified in many ways. Special functional forms may be exploited to create tight convex relaxations. Ideally, we are interested in generating the *convex envelope* [52] of a function in *X*, i.e., the tightest possible convex function which is still an underestimator of *f*. However, despite good progress [52] a technique to obtain the convex envelope of a general non-linear expression has not yet been invented, which is one of the reasons that make problems of type *P* difficult to solve.

Because the tightest possible underestimators may not be calculated in the general case (or even in many special cases), specialised techniques are used to underestimate particular functional forms, and the quality of underestimation is commonly quantified using the maximum separation distance between the original function and its relaxation [5, 20].

Convexification techniques for specific functional forms have multifarious manifestations; for instance, McCormick [34] proposed a strategy to generate convex relaxations of factorable functions. Maranas and Floudas [33] derived tight relaxations for trilinear and fractional functions, while Meyer and Floudas [35] derived convex envelopes for edge-concave functions. Zorn and Sahinidis [60] used a combination of reformulation–linearization [46] and convex envelope construction techniques [53, 54] to produce tight formulations for underlying bilinear structures. Misener and Floudas [37] developed piecewise-linear approximations of multilinear functions for dimension $N\le 3$. Mitsos et al. [38] and Scott et al. [45] proposed the generation of generalised McCormick relaxations to address more general problems. The reader is referred to the review of Floudas and Gounaris [19] for more examples of relaxations of NLPs.

Although specialised techniques may be very effective when particular mathematical structure is present, practical problems manifest in infinitely many mathematical configurations. Thus, general methods to underestimate these problems are needed. Such methods include: (1) directed acyclic graph representations where expressions are partitioned into their component parts using auxiliary variables [9, 28, 49, 52, 54], (2) propagation of subgradients to recursively relax expressions [38, 45], and (3) the $\alpha$BB  [5, 32] general underestimation methodology.

Among these options, the $\alpha$BB methodology and McCormick relaxation methodologies exhibit a useful combination of properties: (1) they may be used to underestimate any $C^2$ function (including trigonometric functions), and (2) they are proven to possess a quadratic convergence rate [10].

Out of these properties, the latter is particularly useful: general non-linear problems may be solved to global optimality using other methods as well, such as methods based on interval analysis [21, 39]. However, interval methods suffer from the dependency problem, and an interval containment set is generally not guaranteed to improve after branching [21]. Higher-order interval methods (e.g., [6, 15, 50]) have been proposed which can mitigate these factors at the expense of additional computational cost [6]. $\alpha$BB is an attractive choice to solve problem *P* because its convergence rate is quadratic, regardless of the order of interval arithmetic it employs. Reduction of the maximum separation distance of at least quadratic rate has been shown to be an important component (an other component being the convergence prefactor [56]), in preventing clustering [16, 40, 56] in branch-and-bound algorithms.

The $\alpha$BB underestimator for general non-convex terms works through superposition of a convex function onto the original non-convex term. Given a box-constrained problem, an underestimating function $L:X\rightarrow \mathbb {L}\subset \mathbb {R}$ may be constructed for any general $C^2$ function *f* by superposing it with a sum of univariate quadratics of sufficient magnitude $\alpha _i$ along each basis vector:

3 $$\begin{aligned} L(\varvec{x})=f(\varvec{x})+\sum _{i=1}^N\alpha _i(x_i^L-x_i)(x_i^U-x_i) \end{aligned}$$

where *N* is the number of variables and $\alpha _i=\max (0,-\frac{1}{2}\lambda _i^{\min })$ [2], $\lambda _i^{\min }$ being a valid lower bound on eigenvalue $\lambda _i$ over the underestimation domain. Interval calculations [21, 39] on the expressions of the second derivatives of *f* may be used to derive its *interval Hessian matrix* [21] *H*(*X*) over an interval $X\subseteq X_0$. The range of the eigenvalues of *H*(*X*) can provide guaranteed enclosures for the possible values of the eigenvalues of the scalar Hessian matrix $H(\varvec{x})$, over all points $\varvec{x}\in X$. These enclosures may subsequently be used to perform a rigorous calculation of $\varvec{\alpha }$.

The $\alpha$BB methodology has seen numerous modifications. Akrotirianakis and Floudas [3, 4] proposed $\gamma$BB, an underestimator which is based on exponential functions instead of the standard $\alpha$BB quadratics, which they proved to be at least as tight as $\alpha$BB. Meyer and Floudas [36] produced a refinement of the classical $\alpha$BB underestimator using a piecewise quadratic perturbation function. This perturbation function was shown to be able to produce significantly tighter underestimators than $\alpha$BB because, unlike classical $\alpha$BB, it can be non-convex. Skjal et al. [48] generalised the classical $\alpha$BB perturbation methodology using the non-diagonal $\beta _{ij}$ elements of the perturbation Hessian matrix. This method comes at relatively small computational overhead, as the perturbation Hessian can be found by solving a linear problem, with the resulting underestimators being at least as tight as the original $\alpha$BB ones. Skjal and Westerlund [47] and Guzman et al. [20] performed computational studies benchmarking the performance of commonly used components in the $\alpha$BB framework, concluding that, for their test sets, the classical $\alpha$BB algorithm using the scaled Gerschgorin theorem [2] is a balanced choice for a default configuration.

However, the advantages of the $\alpha$BB functional form come at a price. Intuitively, because the underestimator is produced by adding another function to the original one, $\alpha$BB may reach a maximum separation distance limit for certain geometries. Since the second, convexifying function is always a sum of univariate quadratics, there must be cases where that sum is as tight as possible, and may not be tightened further without compromising convexity, or changing the functional form of the underestimator.

In this paper we formalise this intuition by deriving the conditions for which an $\alpha$BB underestimator may yield an exact lower bound over a general domain, and provide the theoretical foundation for the GLOBIE algorithm [23]. Given the theoretical certainty that the underestimator will not give an exact lower bound under certain predictable conditions, even for very small boxes, the purpose of this work is to demonstrate that it is theoretically possible to overcome this theoretical limit through mathematical manipulation. We prove this concept by constructing a methodology which allows a priori control over the maximum separation distance between the original function and the convex underestimator in some domains that do not contain the global solution.

Specifically, we propose the transformation of *f* to a modified *subenergy* ($\mu$-*subenergy*) function [8, 12], originally proposed as a tunnelling [27, 58] technique, and demonstrate that, provided exact bounds on the eigenvalues can be obtained, our method may produce arbitrarily tight $\alpha$BB underestimators and fathom nodes in the branch-and-bound tree in cases where it is otherwise theoretically impossible to do so using the classical $\alpha$BB methodology. The purpose of this work is to prove that it is possible to achieve arbitrarily tight $\alpha$BB underestimators, but we would like to stress that this is a theoretical proof-of-concept: in practice, there is no way to obtain exact eigenvalue bounds for a general function, and therefore we investigate the computational behaviour of the approach based on eigenvalue sampling, similar to [17, 57], in some domains that do not contain the global solution. The results of this investigation are in line with our theoretical predictions, as using this heuristic approach we are able to fathom nodes which may not be fathomed using classical $\alpha$BB. In fact, this is the first method that we are aware of which may theoretically produce a convex relaxation of arbitrary tightness (i.e., where the underestimator is arbitrarily close to the original function) for a general non-convex function in some domains. Our work highlights the benefits of producing exact lower bounds by post-processing underestimators (e.g., using mathematical transformations), and suggests two avenues of research: the formulation of such transformations, and the invention of methods to derive rigorous values of $\varvec{\alpha }$ without the use of interval analysis.

This paper is structured as follows: in Sect. 2 we derive the necessary conditions to get exact lower bounds using $\alpha$BB. In Sect. 3 we introduce the $\mu$-subenergy function and discuss its theoretical properties. In Sect. 4 we use this function in conjunction with $\alpha$BB to produce tight convex relaxations, and then apply our method to a step-by-step example in Sect. 5. This is followed by numerical experiments on some well known test functions in Sect. 6, and our conclusions in Sect. 7.

## Dependence of $\alpha$BB underestimators on function concavity

### Preliminaries

Before proceeding, we introduce key notation. Given the optimization problem *P* described in Eq. (1), function $f:X_0\rightarrow F_0\subset \mathbb {R},\,X_0=\{\varvec{x}:\varvec{x}\in [\varvec{x}^L,\varvec{x}^U]\}\subset \mathbb {R}^N$, is a general $C^2$ function. The $\alpha$BB underestimator is defined over a box, therefore we assume that each node in the branch-and-bound tree is a box.

The domain $X_0$ is defined to be the root node, while a sub-domain $X\subseteq X_0$ refers to an arbitrary node *X* during the process of a branch-and-bound tree (which may also be the root). The set $V=\{v_1,v_2,\ldots ,v_{N_{vertices}}\} \subset X$ refers to the set of vertices of the box.

A value $f^*\in F_0$ refers to an arbitrary value of *f*, but in practice it is preferred to be the *best upper bound* (BUB) across the entire branch-and-bound tree, and $\varvec{x}^*\in X_0$ is a point such that $f(\varvec{x}^*)=f^*$.

A sub-domain of $$ X_0 $$ will be referred to as *sub-optimal* with respect to the current value of $$ f^* $$, if *f* acquires values greater than $$ f^*$$ everywhere in that sub-domain.

A value $f^\dagger \in F_0$ is the function value at a global minimum $\varvec{x}^\dagger$ over the whole domain $X_0$.

The value at the global minimum $\varvec{x}^m$ of *f* in a sub-domain *X* is denoted as $f^m\ge f^\dagger ,~f^m\in F\subseteq F_0$.

### Motivation

Consider a convex $\alpha$BB underestimator *L* of a general function *f*, over some domain $X\subseteq X_0\subset \mathbb {R}^N$ where *f* is non-convex. The tightest possible form of this underestimator may be calculated if exact eigenvalue bounds ($\lambda _i^{\min }, i = 1, ..., N$) are known. Because *L* is the sum of a convex function and the original function *f*, the distance between the minimum value of *f* and that of *L* in *X* will always be strictly greater than zero unless the minima of *f* over *X* lie on the vertices of the box. If the minimum in node *X* does lie on a vertex, it is interesting to derive the conditions for which getting an exact lower bound using $\alpha$BB is theoretically possible. In order to motivate this approach, consider the following optimization problem:

4 $$\begin{aligned} \underset{x\in X_0}{\min }~f(x)=\sin (5x)+x^2+2,\,X_0=[-\,2,2] \end{aligned}$$

After rounding down all results at the second decimal, the solution $x^\dagger \approx -0.29$ of this problem yields an objective value of $f^\dagger =f(-0.29)\approx 1.09$. Now consider that, during the course of a branch-and-bound tree, we wish to formulate the lower bounding problem for node $X=[-2,-1]$. The minimum value of the second derivative of *f* in *X* may be derived analytically, i.e., $\underline{f}''(X)=\lambda ^{\min }=f''(-1)=2-25\sin (5(-1))\approx -21.97\Rightarrow \alpha \approx 10.99$ (rounded up), forming an $\alpha$BB relaxation of the original function:

5 $$\begin{aligned} \underset{x\in [-2,-1]}{\min }L(x)=\underbrace{\sin (5x)+x^2+2}_{f(x)}~~ +\underbrace{10.99(x+2)(x+1)}_{\text {convex quadratic perturbation}~q(x)} \end{aligned}$$

**Fig. 1:**
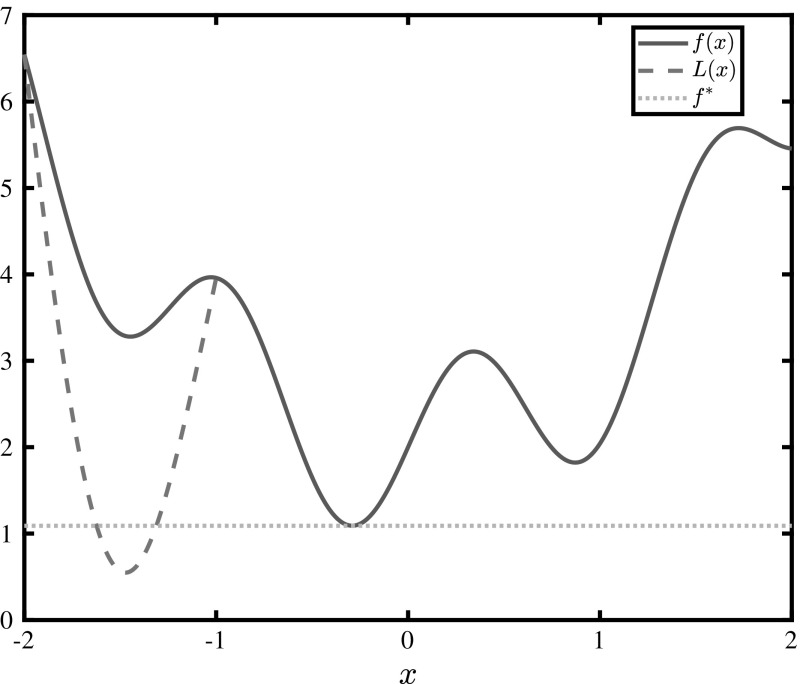
Illustration of the tightest possible $\alpha$BB underestimator *L*(*x*) of $f(x)=\sin (5x)+x^2+2,x\in [-2,-1]$. It is impossible to fathom this region using $\alpha$BB  without branching.

This problem is illustrated in Fig. 1. Note that even if the value at the global minimum $f^\dagger \approx 1.09$ is known, domain *X* may not be fathomed without branching anew.

The $\alpha$BB underestimator convexifies *f* by adding enough convexity to compensate for the worst-case scenario of non-convexity. The quadratic, which has a constant second derivative throughout X, compensates for this by adding constant curvature at every point in the interval. In node *X*, this scenario is the minimum value of the second derivative of *f* in *X*, at $x=-1$.

This convexification, however, comes at a price: the relaxation will only match the original function at the vertices of the box *X*, forcing a gap between the two functions everywhere else. For instance, at the minimum of *f* in *X*, *q* adds $q(-1.44)\approx -2.71$ making the result such that the node may not be fathomed. It can be proven that this can not be avoided: if a smaller $\alpha$ is used, there will be at least one point in *X* where *L* is non-convex. Furthermore, because *f* is non-convex in *X*, $\alpha$ *must* be non-zero. Thus, $\min _{x\in X}(f(x))>\min _{x\in X}(L(x))$, unless the optimal solution lies on one of the vertices $v\in V$ of the box, where it is possible for these two minima to coincide.

#### Theorem 1

If *f* is non-convex over a non-singleton domain $$X\subseteq X_0$$ and a unique global minimum exists in *X*, an $$\alpha $$BB underestimator may only yield the minimum value $$f^m=\underset{\varvec{x}\in X}{\min }f(\varvec{x})$$ if the global solution $$\varvec{x}^m\in X$$ of $$\underset{\varvec{x}\in X}{\min }f(\varvec{x})$$ which corresponds to $$ f^m$$ lies on a vertex $$ v\in V $$ of *X*.

#### Proof

Assume that $\varvec{x}^m$ is not a vertex of the box *X*. Then, at $\varvec{x}=\varvec{x}^m$:

6 $$\begin{aligned} L(\varvec{x}^m)=f(\varvec{x}^m)+\sum _{i=1}^{N}\alpha _i(x_i^L-x_i^m)(x_i^U-x_i^m) \end{aligned}$$

*f* is non-convex over *X*, therefore $\alpha _i>0, \forall i=1, \ldots ,N$. Thus, the quadratic perturbation in this equation is strictly negative if $\varvec{x}^m$ does not belong to the set of vertices *V*. This means that if $\varvec{x}^m\notin V$, $L(\varvec{x}^m)<f(\varvec{x}^m)$.

#### Theorem 2

If *f* is non-convex over $X\subseteq X_0$, its minimum $\varvec{x}^m$ lies on a vertex $v\in V \subset X$, and if $x^L_i\ne x^U_i$, ${\min }_{\varvec{x}\in X}\,L(\varvec{x})=f(\varvec{x}^m) \Leftrightarrow \alpha _i\le \frac{\nabla _{x_i} f |_{x^m}}{x_i^U+x_i^L-2x_i^m}$, $i=1,\ldots ,N$.

#### Proof

Because *L* is convex, if it has a stationary point in *X* then its minimum value must be at this particular point $\varvec{x}^m$. We discern two cases when the optimum of *f* belongs in *V*: (1) *L* has a stationary point in *X*, and (2) it does not. For each case, we show that the relation in the theorem holds.

1. There is a stationary point. Then, if $\varvec{x}^m\in V$:
  7 $$\begin{aligned} \nabla L|_{\varvec{x}^m} = 0 \Leftrightarrow&\nabla _{x_i} f |_{x^m}+\alpha _i(2x_i^m-x_i^U-x_i^L)=0,\quad \forall i=1, \ldots , N \nonumber \\ \Leftrightarrow&\alpha _i=\frac{\nabla _{x_i} f |_{x^m}}{x_i^U+x_i^L-2x_i^m},~\forall i=1,\ldots ,N \end{aligned}$$
  Note that $\alpha _i$ is always defined because the denominator is equal to $x_i^U+x_i^L-2x_i^m=0$ only at the midpoint *M* of *X*. Since $x_i^L\ne x_i^U$ ($M\notin V$ by definition), the denominator cannot be equal to zero for any $\varvec{x}^m$.
2. There is no stationary point in *X*. In this case, for the minimum to lie at a vertex, *L* must vary monotonically in each $x_i$, as per the rules of monotonicity over an interval (otherwise there would be a stationary point). Therefore, the partial derivative of *L* with respect to any $x_i$ is non-zero. We examine the value of $\alpha _i$ for the different signs of the gradient of *L* with respect to $x_i$ at a vertex:
  1. $\nabla _{x_i}L|_{\varvec{x}^m}>0$. If the function is increasing, then $x_i^m=x_i^L$. Then:
    8 $$\begin{aligned} x_i^U> x_i^L\Leftrightarrow&x_i^U-x_i^L> 0 \nonumber \\ \Leftrightarrow&x_i^U+x_i^L-2x_i^L> 0 \nonumber \\ \Leftrightarrow&2x_i^m-x_i^U-x_i^L< 0 \end{aligned}$$
    Now that the sign of this expression is known, the value of $\alpha _i$ may be bounded from above:
    9 $$\begin{aligned} \nabla _{x_i} L |_{x^m}>0\Leftrightarrow&\nabla _{x_i} f |_{x^m}+\alpha _i(2x_i^m-x_i^U-x_i^L)>0\nonumber \\ \Leftrightarrow&\alpha _i<\frac{-\nabla _{x_i} f |_{x^m}}{2x_i^m-x_i^U-x_i^L}\nonumber \\ \Leftrightarrow&\alpha _i<\frac{\nabla _{x_i} f |_{x^m}}{x_i^U+x_i^L-2x_i^m} \end{aligned}$$
  2. $\nabla _{x_i}L|_{\varvec{x}^m}<0$. Similar to before, if the function is decreasing then $x_i^m=x_i^U \Leftrightarrow 2x_i^m-x_i^U-x_i^L>0$. Then:
    10 $$\begin{aligned} \nabla _{x_i}L|_{\varvec{x}^m}<0\Leftrightarrow&\nabla _{x_i} f |_{x^m}+\alpha _i(2x_i^m-x_i^U-x_i^L)<0\nonumber \\ \Leftrightarrow&\alpha _i<\frac{-\nabla _{x_i} f |_{x^m}}{2x_i^m-x_i^U-x_i^L}\nonumber \\ \Leftrightarrow&\alpha _i<\frac{\nabla _{x_i} f |_{x^m}}{x_i^U+x_i^L-2x_i^m} \end{aligned}$$

which is the final condition to prove the original statement. $\square$

It follows that, even if all interval calculations on the curvature of *f* in *X* are exact, it is impossible to get an exact lower bound on the minimum value of the objective function using a classical $\alpha$BB underestimator, unless the solution of problem $\breve{P}$ satisfies Theorems 1 and 2 in that box. This means that if any of the $\alpha _i$ components is greater than $\frac{\nabla _{x_i} f |_{x^m}}{x_i^U+x_i^L-2x_i^m}$ in a box, it is impossible to get that exact lower bound regardless of whether the minimum lies on a vertex or not. In fact, if the function has highly negative eigenvalues in some parts of the region, the separation distance can be very large, even with exact eigenvalue calculations, as inferred by the maximum separation distance [5] ($d_{\max }$) formula:

11 $$\begin{aligned} d_{\max }=\frac{1}{4}\sum \limits _{i=1}^{N}\alpha _i(\varvec{x}^L,\varvec{x}^U)(x_i^U-x_i^L)^2 \end{aligned}$$

A simple, yet naive, way to reduce this distance would be to scale the entire problem by some factor $K\in (0,1)$, because scaling the underestimator *L* would also scale its eigenvalues:

12 $$\begin{aligned} L_K(\varvec{x})=Kf(\varvec{x})+K\sum \limits _{i=1}^{N}\alpha _i(x_i^L-x_i)(x_i^U-x_i) \end{aligned}$$

It follows from Eq. (12) that, as $K\rightarrow 0$ the maximum separation distance $Kf(\varvec{x})-L_K(\varvec{x})$ between the new $\alpha$BB underestimator and the scaled function also goes to 0. This naive approach may not, of course, yield any benefit. The functions are scaled uniformly, therefore the relative distances between all points of the hypersurface remain the same, i.e., the geometrical shape of the hypersurface and its underestimator is exactly the same, only scaled down. This means that even if the function in Fig. 1 is scaled down by a factor of $K=10^{-3}$, the lower bound $f^*=K\cdot 0.55$ (where 0.55 is the value at the solution of *L*(*x*) in $X=[-2,-1]$) obtained through exact $\alpha$BB underestimation of domain *X* is still not large enough to fathom node *X*, when compared to the scaled global minimum $f^\dagger =K\cdot 1.09$.

Although this simple approach does not allow any improvement, Eq. (12) illustrates a mechanic which may be exploited: the maximum separation distance using the $\alpha$BB functional form may become arbitrarily small by scaling down the problem.

#### Hypothesis 1

If a function may be scaled down in a non-uniform way, such that $L_K(\varvec{x})\rightarrow 0$ in some region *X* that does not contain the global solution, but in the neighbourhood of the global solution $|Kf^\dagger |\ge \epsilon ,\,\epsilon \in \mathbb {R}^+$, and at the same time all points in the new function maintain their relative positions to each other, the new $\alpha$BB underestimator over $X\subset X_0$ may then be tight enough to enable fathoming *X*, even if *f* does not satisfy Theorems 1 and 2 over *X*.

A non-uniform transformation of this type, called the *subenergy function*, was proposed by Cetin et al. [8, 12] as part of a tunnelling [27, 58] technique.

## The $\mu$-subenergy function

The subenergy function of a $C^2$ function *f* is defined as follows [8]:

13 $$\begin{aligned} E(\varvec{x};f^*,a)=\ln \left( \frac{1}{1+e^{-(f(\varvec{x})-f^*)-a}}\right) \end{aligned}$$

where $f^*,a$^1^ are parameters. For the purpose of this work, this function is modified such that it is bounded from above by a logarithmic barrier at 0. This step is necessary to give the function useful bounding properties because it fixes the value range of the subenergy function. We will refer to the modified version of this function as the $\mu$-subenergy function, defined as follows:

14 $$\begin{aligned} S(\varvec{x};f^*,\mu )=-\ln (1+e^{-\mu (f(\varvec{x})-f^*)}) \end{aligned}$$

where $f^*$ and $\mu \in \mathbb {R}^+$ are parameters. Parameter $\mu$ will be referred to as the *subenergy magnitude*; we will show that by appropriate choice of this magnitude it is possible to impose a number of desirable bounding properties on the $\mu$-subenergy function.

### Properties of the $\mu$-subenergy function

In order to perform an analysis on the properties of the new function, the following definitions need to be introduced:

#### Definition 1

A function $f:X_0\rightarrow F_0\subset \mathbb {R},\,X_0\subset \mathbb {R}^N$, is said to be *subenergy flattened* (or *flattened, for brevity*) across $X_1\subset X_0$, if the function is mapped onto a $\mu$-subenergy function *S* with the following properties: (1) the eigenvalues of the Hessian matrix of $S(\varvec{x};f^*,\mu )$ are of smaller magnitude than those of the Hessian matrix of $f(\varvec{x})~\forall \varvec{x}\in X_1$, and (2) *S* has the same monotonicity and stationary points as *f*. A function which has been flattened will be referred to as *flat* over $X_1$, and the property of having been flattened will be referred to as *flatness*.

#### Definition 2

A $\mu$-subenergy function is said to become *flatter* in $X_1$ as the magnitude of each of the eigenvalues of its Hessian matrix in that domain gets closer to zero.

Intuitively, if *f* may be transformed such that the transformation is flatter in some domains, but not flat in others, $\alpha$BB underestimators in the flat domains will produce proportionally smaller maximum separation distances than in the less flat ones, which would satisfy Hypothesis 1.

**Fig. 2:**
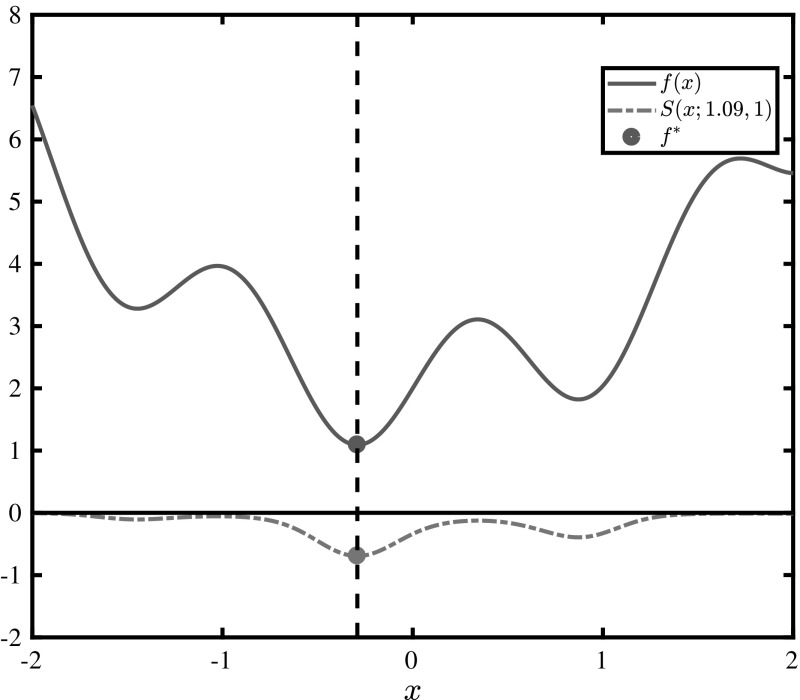
Illustration of the $\mu$-subenergy transformation of $f(x)=\sin (5x)+x^2+2$ for $f^*=1.09$ and $\mu =1$

An example of a $\mu$-subenergy transformation is illustrated in Fig. 2, for $f^*=f^\dagger$. The new function appears to be non-uniformly smoother, i.e, the magnitude of its curvature is smaller at its maxima, and the monotonicity and stationary points are invariant with respect to the transformation. This is a fundamental property of the $\mu$-subenergy function, i.e., it is a continuous bijective transformation of the original objective function that does not preserve curvature but preserves monotonicity and stationary points. These properties are established in Theorem 3:

#### Theorem 3

Let $f:X\rightarrow F\subseteq F_0\subset \mathbb {R},\, X\subseteq X_0\subset \mathbb {R}^n$ be a $C^2$ function, $\mu \in \mathbb {R}^+$ be a constant, and $S(\varvec{x};f^*,\mu )=-\ln \left( 1+e^{-\mu (f(\varvec{x})-f^*)}\right) ,\,\varvec{x}\in X$ be its $\mu$-subenergy function, where $f^*\in F_0$ is an arbitrary value for some $\varvec{x} \in$ $X_0$. Then, *S* and *f* have the same monotonicity and stationary points.

#### Proof

Consider a partial derivative of $S(\varvec{x};f^*,\mu )$, with respect to an arbitrary $x_i$:

15 $$\begin{aligned} \frac{\partial }{\partial {x_i}}S(\varvec{x};f^*,\mu )= \frac{\mu }{1+e^{\mu (f(\varvec{x})-f^*)}}\frac{\partial }{\partial {x_i}}f(\varvec{x}) \end{aligned}$$

Let $A=\frac{\mu }{1+e^{\mu (f(\varvec{x})-f^*)}}$. Function *f* is bounded, which means that $f(\varvec{x})-f^*\ne \pm \infty$. This means that the expression $\frac{1}{1+e^{\mu (f(\varvec{x})-f^*)}}\in (0,1)$, since the denominator is always strictly greater than 1. Multiplying this expression by $\mu$ yields:

16 $$\begin{aligned} 0<\frac{\mu }{1+e^{\mu (f(\varvec{x})-f^*)}}<\mu \end{aligned}$$

Hence, $A\in (0,\mu )$. Since *A* is always positive, all first derivatives of *S* and *f* have the same sign $\forall \varvec{x}\in X$. Furthermore, by Eq. (15) and $A>0$:

17 $$\begin{aligned} \frac{\partial }{\partial {x_i}}f(\varvec{x})=0\Leftrightarrow \frac{\partial }{\partial {x_i}}S(\varvec{x};f^*,\mu )=0 \end{aligned}$$

Therefore, the two functions have the same monotonicity as well as stationary points. $\square$

#### Corollary 1

The $\mu$-subenergy function preserves the relative positions of the image of every point of the original function, including the global minimum. In other words, $f(\varvec{x}_1)\ge f(\varvec{x}_2)\Leftrightarrow S(\varvec{x}_1;f^*,\mu )\ge S(\varvec{x}_2;f^*,\mu ),\forall \varvec{x}_1,\varvec{x}_2\in X$, including ${\varvec{x}}$, $\varvec{x}^\dagger :$ $f(\varvec{x})\ge f^\dagger \Leftrightarrow S(\varvec{x};f^*,\mu )\ge S(\varvec{x}^\dagger ;f^*,\mu )$.

#### Remark 1

Since the relative positions of all points are preserved, all local extrema are preserved as well.

#### Lemma 1

The $\mu$-subenergy function acquires a value of $S(\varvec{x};f^*,\mu )=-\ln (2)$ if and only if $\varvec{x}=\varvec{x}^*$.

#### Proof

$$\varvec{x}=\varvec{x}^*\Leftrightarrow S(\varvec{x};f^*,\mu )=-\ln (1+e^0)=-\ln (2)$$. $$\square $$

Note that, as a consequence of Corollary 1, the global minimizer $\varvec{x}^\dagger$ is preserved, i.e., it is also the global minimizer of *S*. Hence, by Lemma 1, if $f^*=f^\dagger$ the value of the $\mu$-subenergy function at the global minimum is $S(\varvec{x}^\dagger ;f^\dagger ,\mu )=-\ln (2)$. This observation is very important because it indicates that, if $f^*=f^\dagger$, the global minimum will attain a constant value, regardless of the flatness of the $\mu$-subenergy function in other sub-domains. Subenergy values are straightforward to map back to the *f*-space, as shown in Lemma 2.

#### Lemma 2

For all $\varvec{x}\in X_0$, any value in the *S*-space may be mapped back to the *f*-space using the reverse $\mu$-subenergy transformation:

18 $$\begin{aligned} f(\varvec{x})=-\frac{1}{\mu }\ln (e^{-S(\varvec{x};f^*,\mu )}-1)+f^* \end{aligned}$$

#### Proof

For all $$ \varvec{x}\in X_0 $$:

19 $$\begin{aligned} S(\varvec{x};f^*,\mu )=-\ln (1+e^{-\mu (f(\varvec{x})-f^*)})&\Leftrightarrow e^{-S(\varvec{x};f^*,\mu )}-1=e^{-\mu (f(\varvec{x})-f^*)}\nonumber \\&\Leftrightarrow -\ln (e^{-S(\varvec{x};f^*,\mu )}-1)=\mu (f(\varvec{x})-f^*)\nonumber \\&\Leftrightarrow f(\varvec{x})=-\frac{1}{\mu }\ln (e^{-S(\varvec{x};f^*,\mu )}-1)+f^* \end{aligned}$$

$$\square $$

It has been established that the $\mu$-subenergy mapping preserves all local extrema, and it is known that each point is non-uniformly mapped to the new hypersurface, therefore the next step is to derive bounds on the new values after the mapping (Hypothesis 1). In order to do so, it is necessary to first give the following definition of a special case of sub-optimality:

#### Definition 3

For some $f_1>f^*$, sub-domain $X_1=\{\varvec{x}\in X_0:f(\varvec{x})\ge f_1\}$ is defined as $f_1$-sub-optimal with respect to problem *P*.

The concept of $$ f_1$$-sub-optimality is central to the analysis that follows. In Fig. 2 we see that the value of the $$\mu $$-subenergy function is $$ -\ln (2) $$ at $$ x=x^* $$, and that it approaches the logarithmic barrier of 0 as the function acquires values greater than $$ f^* $$. This is a sign of desired behaviour: the function becomes flat at values sufficiently far from $$ f^* $$. In the general case however, the function may not be visualised, therefore this raises the question of what happens to the points which correspond to the values in-between, i.e., whether it is possible to bound the value range of the original function in certain sub-domains of *X*, in their respective $$\mu $$-subenergy-domains. Specifically, it is desired to bound these values within a predictable value range, between some predefined tolerance $$ -\epsilon $$ and 0. In order to answer this question, the concept of $$ f_1 $$-sub-optimality is introduced.

Assume a user-defined constant tolerance $c\in \mathbb {R}^+, c=f_1-f^*$ that defines a distance between $f^*$ and $f_1$, and a tolerance $\epsilon$ in the *S*-space such that $\epsilon \in (0,\ln (2))$. It is possible to derive a minimum value of $\mu$ such that all points with values greater than $f_1$ in *f*-space are mapped between two barriers in *S*-space: $-\epsilon$ and 0. In other words, $f_1$ in *f*-space may be mapped to $-\epsilon$ in *S*-space, and all values greater than *c* may be mapped to values greater than $-\epsilon$. This gives the user control over the magnitude of *S* in $f_1$-sub-optimal domains.

#### Theorem 4

Any $f_1$-sub-optimal point $\varvec{x}$ (such that $f(\varvec{x})\ge f_1$) is mapped within $[-\epsilon ,0)$, by the transformation *S* with parameters $\mu$ and $f^*$, iff $\mu \ge -\frac{\ln (e^{\epsilon }-1)}{c}$, where $\epsilon \in (0,\ln (2)),c=f_1-f^*$.

#### Proof

Let $$\varvec{x}_1\in X_1:S(\varvec{x}_1;f^*,\mu )\ge -\epsilon $$ and $$f({\varvec{x}}_1) = f_1>f^*$$:

20 $$\begin{aligned} -\ln (1+e^{-\mu (f_1-f^*)})\ge -\epsilon&\Leftrightarrow 1+e^{-\mu (f_1-f^*)}\le e^{\epsilon }\nonumber \\&\Leftrightarrow e^{-\mu (f_1-f^*)}\le e^{\epsilon }-1\nonumber \\&\Leftrightarrow -\mu (f_1-f^*)\le \ln (e^{\epsilon }-1) \nonumber \\&\Leftrightarrow \mu \ge -\frac{\ln (e^{\epsilon }-1)}{f_1-f^*}=-\frac{\ln (e^{\epsilon }-1)}{c} \end{aligned}$$

$$\square $$

Because of these bounding properties, and because the $\mu$-subenergy transformation appears to make the function more flat in certain sub-domains, it is of interest to investigate possible advantages of calculating the $\alpha$BB relaxation of the $\mu$-subenergy function and subsequently mapping the resulting lower bound from *S*-space to *f*-space. The success or failure of such an endeavour is linked to the following items: (1) the effect of varying the two parameters, $f^*$ and $\mu$ (before and while a branch-and-bound tree is in progress), and (2) how these parameters affect the derivatives and the eigenvalues of the Hessian matrix of the $\mu$-subenergy function. In particular, it is desired to derive a link between these parameters and the bounds on the eigenvalues of the Hessian matrix of the $\mu$-subenergy function in flat sub-domains, because eigenvalues directly affect $\alpha$BB lower bounds. However, before investigating this link, the treatment of the $\mu$-subenergy parameters in a branch-and-bound process must be discussed.

### The effect of varying the subenergy parameters $f^*$ and $\mu$

Having defined the mathematical significance of $\mu$, $f_1$, *c*, and $\epsilon$, we may now investigate how the variation of the $\mu$ and $f^*$ parameters affects the transformation, particularly its lower bounds and flatness. Furthermore, in order to be able to use the $\mu$-subenergy function with $\alpha$BB in a branch-and-bound context, we determine whether $\mu$ and $f^*$ should be allowed to acquire different values between different nodes in a branch-and-bound tree, i.e., whether a lower bound derived using a particular combination of $\mu$ and $f^*$ is still valid if a different combination is used elsewhere in the tree.

#### Effect of $f^*$

**Fig. 3:**
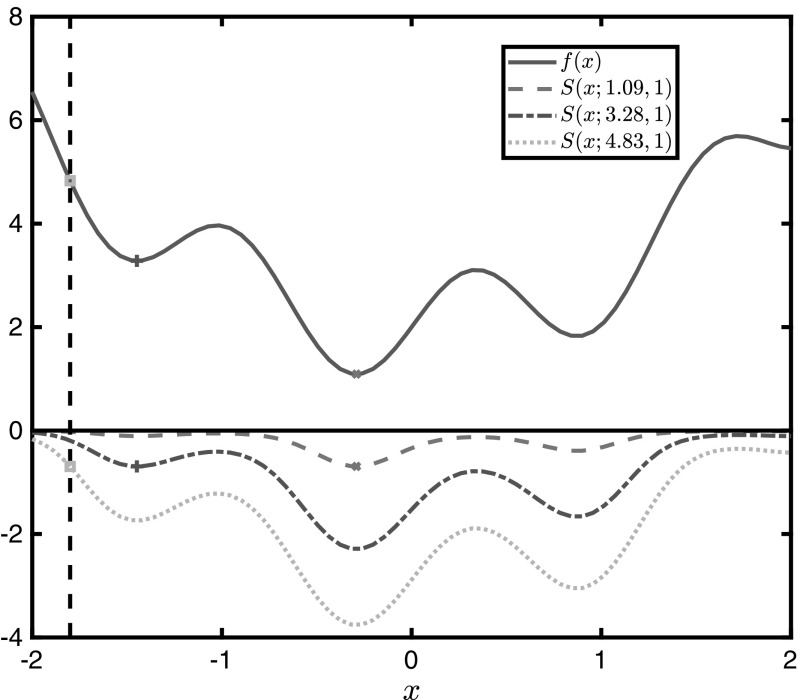
Illustration of the $\mu$-subenergy transformation of $f(x)=\sin (5x)+x^2+2$ for three different values of $f^*$

The effect of varying $f^*$ is displayed in Fig. 3, for a fixed value of $\mu =1$. It may be observed that, as better (smaller) $f^*$ are discovered, sequentially increasing subsets of $X_0$ become flatter and are mapped between two natural barriers: $-\ln (2)<S(\varvec{x};f^*,\mu )<0$. Ideally, it is desired to always use $f^*=f^\dagger$ because it translates into flattening *f* over the largest possible subset of the solution space, for a given $\mu$. However, in modern algorithms which make use of convexification techniques, the optimal objective value is rarely available from the beginning, and upper bounds are improved incrementally.

##### Theorem 5

Let $f^*_1,f^*_2$ be arbitrary values of *f* in $X_0$, such that $f^*_1>f^*_2$. Then, $\forall \varvec{x}\in X_0$, $S(\varvec{x};f^*_{1},\mu )<S(\varvec{x};f^*_{2},\mu )\Leftrightarrow f^*_{1}>f^*_{2}.$

##### Proof

$f^*_{1}>f^*_{2}\Leftrightarrow -\mu (f(\varvec{x})-f^*_{1})>-\mu (f(\varvec{x})-f^*_{2}),\,\forall \varvec{x}\in X_0$. The exponential function is monotonically increasing, so, $\forall \varvec{x}\in X_0$, $-\mu (f(\varvec{x})-f^*_{1})>-\mu (f(\varvec{x})-f^*_{2})\Leftrightarrow e^{-\mu (f(\varvec{x})-f^*_{1})}>e^{-\mu (f(\varvec{x})-f^*_{2})}.$ The subenergy magnitude is always positive, therefore $\forall \varvec{x}\in X_0$:

21 $$\begin{aligned} e^{-\mu (f(\varvec{x})-f^*_{1})}>e^{-\mu (f(\varvec{x})-f^*_{2})}\Leftrightarrow & {} 1+e^{-\mu (f(\varvec{x})-f^*_{1})}>1+e^{-\mu (f(\varvec{x})-f^*_{2})}\nonumber \\\Leftrightarrow & {} \ln (1+e^{-\mu (f(\varvec{x})-f^*_{1})})>\ln (1+e^{-\mu (f(\varvec{x})-f^*_{2})})\nonumber \\\Leftrightarrow & {} -\ln (1+e^{-\mu (f(\varvec{x})-f^*_{1})})<-\ln (1+e^{-\mu (f(\varvec{x})-f^*_{2})})\nonumber \\\Leftrightarrow & {} S(\varvec{x};f^*_{1},\mu )<S(\varvec{x};f^*_{2},\mu ) \end{aligned}$$

$$\square $$

##### Corollary 2

If $f^\dagger$ is the value at a global minimum of *f* over $X_0$, then $S(\varvec{x};f^*,\mu )< S(\varvec{x};f^\dagger ,\mu )$ $\forall \varvec{x}\in \left\{ X_0 \backslash \varvec{x}:f(\varvec{x})=f^\dagger \right\}$.

Assume a current upper bound $f^*_1$ in a branch-and-bound tree, and a new upper bound $f^*_2$, such that $f^*_1>f^*_2$. Further assume some lower bounds $S^L_1$ and $S^L_2$ derived for each of these $\mu$-subenergy instances respectively, using $\alpha$BB underestimators of $S_1(\varvec{x};f^*_1,\mu )$ and $S_2(\varvec{x};f^*_2,\mu )$ respectively. As illustrated in Fig. 3, every time $f^*$ is updated for a smaller value, the old instance of *S* is an underestimator of the new one. In fact, any underestimator of an old instance will be a valid underestimator of any new one, as proven in Theorem 5, provided that $\mu$ remains unchanged. In other words, Theorem 5 guarantees that the lower bound derived from the old instance, $S^L_1$, is still a valid lower bound on the new instance. Therefore, it is safe to update the value of $f^*$ during the branch-and-bound process, and all previously calculated lower bounds using some value of $f^*$ will still be valid if a better (smaller) value is used in future iterations of a branch-and-bound tree.

#### Effect of the subenergy magnitude

**Fig. 4:**
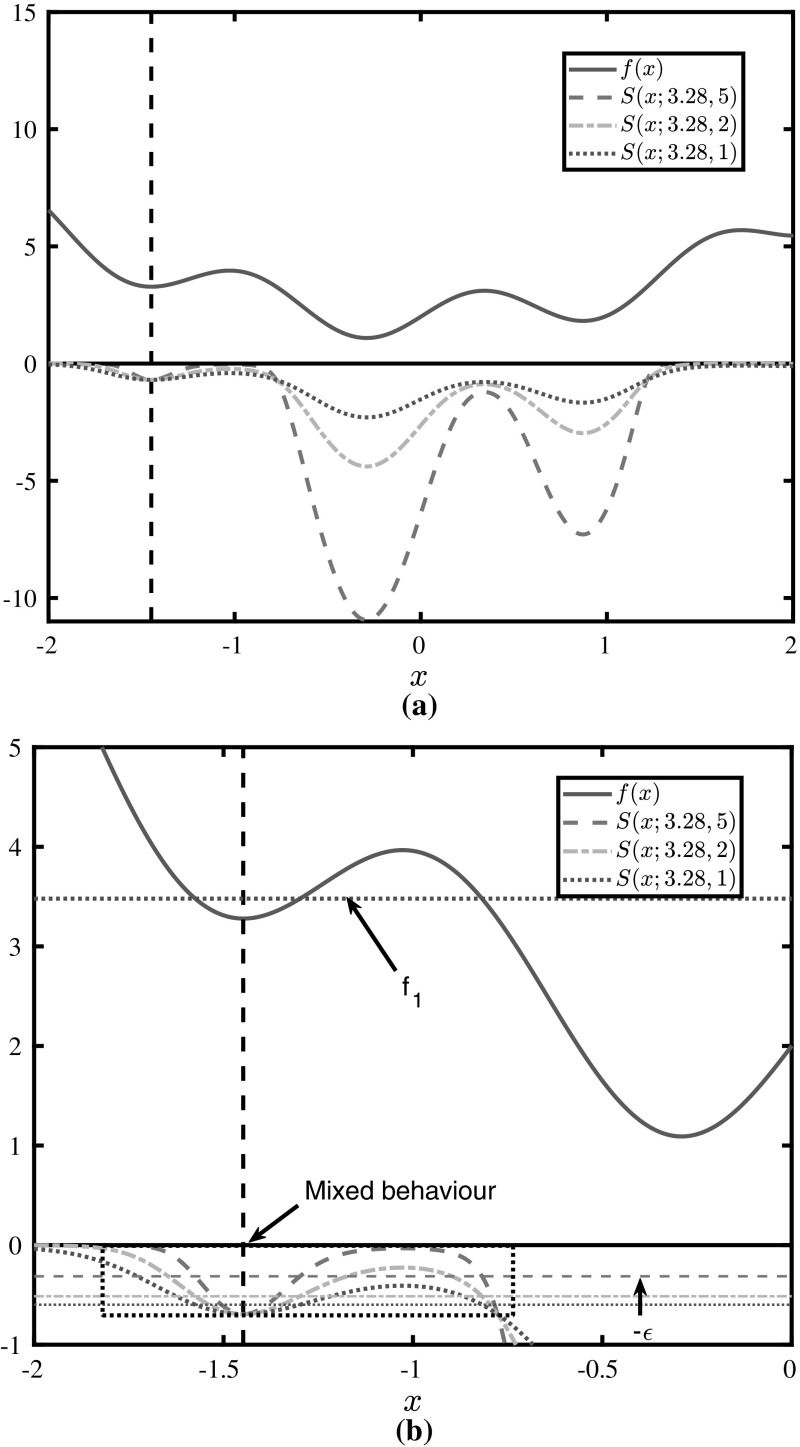
Illustration of the $\mu$-subenergy transformation of $f(x)=\sin (5x)+x^2+2$ for three different values of $\mu$. **a** The $\mu$-subenergy  transformation for varying values of the subenergy magnitude $\mu$. **b** An expanded view of the $\mu$-subenergy transformation in the neighbourhood of $x^*$

The effect of varying the subenergy magnitude is illustrated in Fig. 4a. Greater subenergy magnitudes make the $\mu$-subenergy function flatter across $f_1$-sub-optimal domains, and result in curvature of greater magnitude in optimal domains, i.e., where $f(\varvec{x})\le f^*$. In Sect. 4 it is shown that this flattening effect is guaranteed across $f_1$-sub-optimal domains, while in all other domains the $\mu$-subenergy function may exhibit mixed behaviour (may be flatter or not), as seen in the close-up in Fig. 4b. The extent of this effect may effectively be controlled across all sub-optimal domains because *c* and $\epsilon$ can be chosen such that $X_1\cap X\approx \emptyset$.

Furthermore, Fig. 4 illustrates the result of Theorem 4, where increasing $\mu$ maps *f* closer to zero in the $f_1$-sub-optimal domains. This effect is also observed by examining the monotonicity of Eq. (20) as, for a fixed value of *c*, an increase in $\mu$ brings the $-\epsilon$ barrier closer to 0. Note that, as seen in Fig. 4b, for values of *f* smaller than $f^*$, $\mu$-subenergy lower bounds are not preserved if $\mu$ is changed. Thus, $\mu$ should always be kept constant in order to avoid recalculation of lower bounds.

## Convex relaxation of the $\mu$-subenergy function using $\alpha$BB

### Properties of using exact eigenvalue bounds

Let a convex underestimator be formulated over a domain *X*.

#### Definition 4

An underestimator may have the same optimal value as the original function, without being the tightest possible. In this case, *the bound of the underestimator* is said to be *exact*.

#### Remark 2

If the underestimator is derived using interval arithmetic, and the interval bounds are exact, i.e., they correspond to the true extreme values of the function in that domain, it will generally not provide an exact lower bound, but it will be the tightest possible underestimator for the particular functional form of that underestimator.

An $\alpha$BB underestimator is said to be exact if the $\varvec{\alpha }$ vector is obtained using exact eigenvalue calculations, i.e., the bounds on the eigenvalues of the set of the Hessian matrices of *f* over *X* are exact. In this case, the underestimator is the tightest that may be achieved using the $\alpha$BB functional form. Because $x^*$ may not (by definition) be part of any $f_1$-sub-optimal node, all nodes which are $f_1$-sub-optimal may in principle be fathomed. Therefore, it is of interest to derive bounds on their derivatives in the $\mu$-subenergy-domain.

We begin the analysis of the derivatives of the $\mu$-subenergy function by deriving bounds on the gradient of $S(\varvec{x};f^*,\mu ),~\forall \varvec{x}\in X_1$.

#### Theorem 6

The magnitude of the gradient of $S(\varvec{x};f^*,\mu )$ approaches 0 as $\mu \rightarrow \infty$, $\forall \varvec{x}\in X_1$.

#### Proof

Consider an arbitrary first derivative of *S*:

22 $$\begin{aligned} \frac{\partial }{\partial x_i} S(\varvec{x};f^*,\mu )=\frac{\mu }{1+e^{\mu (f(\varvec{x})-f^*)}}\frac{\partial }{\partial x_i}f(\varvec{x})=A\frac{\partial }{\partial x_i}f(\varvec{x})\end{aligned}$$

We wish to derive a bound on $A=\frac{\mu }{1+e^{\mu (f(\varvec{x})-f^*)}}$, as $\mu \rightarrow \infty$. Because $\varvec{x}\in X_1$:

23 $$\begin{aligned} f(\varvec{x})\ge f_1&\Leftrightarrow f(\varvec{x})-f^*\ge f_1-f^*\nonumber \\&\Leftrightarrow 1+e^{\mu (f(\varvec{x})-f^*)}\ge 1+e^{\mu (f_1-f^*)}\nonumber \\&\Leftrightarrow \frac{\mu }{1+e^{\mu (f(\varvec{x})-f^*)}}\le \frac{\mu }{1+e^{\mu (f_1-f^*)}}\nonumber \\&\Leftrightarrow \frac{\mu }{1+e^{\mu (f(\varvec{x})-f^*)}}\le \frac{\mu }{1+e^{\mu c}} \end{aligned}$$

*A* is always positive, therefore it is bounded from below by 0. By Eq. (23), as $\mu \rightarrow \infty$, *A* is also bounded from above by:

24 $$\begin{aligned} 0\le A\le \lim _{\mu \rightarrow \infty }\frac{\mu }{1+e^{\mu c}}= \lim _{\mu \rightarrow \infty }\frac{1}{c e^{\mu c}}=0 \end{aligned}$$

where de l’Hospital’s Theorem was used. At the limit, because *A* is equal to 0, the subenergy gradient will always go to 0 regardless of the sign or magnitude of $\frac{\partial }{\partial x_i}f(\varvec{x})$ as $\mu \rightarrow \infty$. $\square$

#### Corollary 3

The minimum value of $\mu$ which imposes some arbitrary bound *K* on the magnitude of the gradient of *S* may be calculated by solving the following optimization problem:25


Because Theorem 6 holds, there must exist a $\mu \in \mathbb {R}^+$ which solves $P_K$. Therefore, large values of $\mu$ map not only the $\mu$-subenergy function values, but also its gradient, arbitrarily close to 0. It is thus possible to impose arbitrary bounds on the gradient of *S* over $X_1$, if any bounds on the gradient of *f* are known. Because *f* and, by extent, *S*, are assumed to be $C^2$ differentiable and bounded, it follows that the eigenvalues of *S* also approach zero when the conditions of Theorem 4 are met:

#### Theorem 7

Given that $\mu$ satisfies Theorem 4, the magnitude of all eigenvalues of $S(\varvec{x};f^*,\mu )$ approaches 0 as $\mu \rightarrow \infty$, $\forall \varvec{x}\in X_1$.

#### Proof

Consider an arbitrary second derivative of *S*:

26 $$\begin{aligned} \frac{\partial ^2}{\partial x_i \partial x_j} S(\varvec{x};f^*,\mu )&= \underbrace{\frac{\mu }{1+e^{\mu (f(\varvec{x})-f^*)}}}_{A}\frac{\partial ^2}{\partial x_i \partial x_j}f(\varvec{x})\nonumber \\&\,\;\,\;\,-\underbrace{\frac{\mu ^2 e^{\mu (f(\varvec{x})-f^*)}}{(1+e^{\mu (f(\varvec{x})-f^*)})^2}}_{B} \frac{\partial }{\partial x_i}f(\varvec{x})\frac{\partial }{\partial x_j}f(\varvec{x})\nonumber \\&= A\frac{\partial ^2}{\partial x_i \partial x_j}f(\varvec{x})- B\frac{\partial }{\partial x_i}f(\varvec{x})\frac{\partial }{\partial x_j}f(\varvec{x})\end{aligned}$$

*A* has already been shown to go to 0 as $\mu \rightarrow \infty ,\,\forall \varvec{x}\in X_1$. *B* is rearranged by factoring out $\mu ^2$ and adding and subtracting 1 from the numerator:

27 $$\begin{aligned} B=\mu ^2\left( \frac{1}{1+e^{\mu (f(\varvec{x})-f^*)}}-\frac{1}{(1+e^{\mu (f(\varvec{x})-f^*)})^2}\right) \end{aligned}$$

Now consider the limit of *B* as $\mu \rightarrow \infty$:

28 $$\begin{aligned} \lim _{\mu \rightarrow \infty }B&= \lim _{\mu \rightarrow \infty } \left( \frac{\mu ^2}{1+e^{\mu (f(\varvec{x})-f^*)}}-\frac{\mu ^2}{(1+e^{\mu (f(\varvec{x})-f^*)})^2}\right) \nonumber \\&=\lim _{\mu \rightarrow \infty } \bigg (\frac{2\mu }{(f(\varvec{x})-f^*)e^{\mu (f(\varvec{x})-f^*)}}-\frac{2\mu }{2(1+e^{\mu (f(\varvec{x})-f^*)})(f(\varvec{x})-f^*)e^{\mu (f(\varvec{x})-f^*)}}\bigg )\nonumber \\&=\lim _{\mu \rightarrow \infty } \bigg (\frac{2}{(f(\varvec{x})-f^*)^2e^{\mu (f(\varvec{x})-f^*)}}\nonumber \\&\,\;\;\;\;-\frac{2}{2(f(\varvec{x})-f^*)^2e^{2\mu (f(\varvec{x})-f^*)}+2(f(\varvec{x})-f^*)^2(1+e^{\mu (f(\varvec{x})-f^*)})e^{\mu (f(\varvec{x})-f^*)}}\bigg )\nonumber \\&=0 \end{aligned}$$

where de l’Hospital’s Theorem was used. Since all second derivatives go to 0, so do all eigenvalues of $S(\varvec{x};f^*,\mu ),\,\forall \varvec{x}\in X_1$. $\square$

#### Corollary 4

All components of $\varvec{\alpha }$ for a $\mu$-subenergy function approach 0 continuously for large values of $\mu$ regardless of the curvature of the original function, in $f_1$-sub-optimal domains.

#### Lemma 3

The maximum separation distance between a $\mu$-subenergy function and its $\alpha$BB underestimator can be imposed to be arbitrarily close to 0 in $f_1$-sub-optimal domains.

#### Proof

Let *d* be the maximum separation distance between the $\mu$-subenergy function and its $\alpha$BB underestimator $L_S$. Then, by the formula of Androulakis et al. [5] the maximum separation distance $d_S$ between $S(\varvec{x};f^*,\mu )$ and $L_S$ in the $f_1$-sub-optimal domain is:

29 $$\begin{aligned} d_S = \frac{1}{4}\sum _{i=1}^{N}\alpha _i(x_i^L-x_i^U)^2\le \frac{1}{4}\alpha _{\max }\sum _{i=1}^{N}(x_i^L-x_i^U)^2 \end{aligned}$$

where $\alpha _{\max }=\max \{\alpha _1,\alpha _2, \ldots ,\alpha _N\}$. The maximum separation distance will be arbitrarily small if the right hand-side of Inequality 29 can be shown to be less than or equal to some arbitrary positive tolerance $\epsilon _1$. Then,

30 $$\begin{aligned} \frac{1}{4}\alpha _{\max }\sum _{i=1}^{N}(x_i^L-x_i^U)^2 \le \epsilon _1 \Leftrightarrow \alpha _{\max }\le \frac{4\epsilon _1}{\sum _{i=1}^{N}(x_i^L-x_i^U)^2} \end{aligned}$$

The denominator of Inequality 30 is always positive because $\alpha$BB underestimators are defined over non-singleton domains. By Theorems 7 and Corollary 4, since $\alpha _{\max }$ tends to zero continuously then there must exist some real value of $\mu$ such that Inequality 30 is satisfied. $\square$

Corollary 4 and Lemma 3 follow naturally from Theorem 7 and the functional form of the $\alpha$BB underestimator. As the $\varvec{\alpha }$ vector for *S*, $\varvec{\alpha }_S\rightarrow \varvec{0}$, the quadratic perturbation vanishes, and $L_S(\varvec{x};f^*,\mu )\rightarrow S(\varvec{x};f^*,\mu )$. A direct result of this is that, regardless of the original values of the original $\varvec{\alpha }$ vector (in the *f*-space), these values will approach zero in the *S*-space for the $f_1$-sub-optimal domain.

#### Theorem 8

An underestimator of $f(\varvec{x})$, acquired by the reverse transformation of the $\alpha$BB underestimator of its $\mu$-subenergy function back to *f*-space, can be arbitrarily tight, $\forall \varvec{x}\in X_1$. In other words, if $\epsilon _f$ an arbitrary tolerance, $\exists \mu :d_f\le \epsilon _f\forall \varvec{x}\in X_1$, where $d_f$ is the maximum separation distance between the underestimator acquired through the reverse transformation and $f(\varvec{x})$.

#### Proof

If $f(\varvec{x})$ is detected to be convex over the domain then the maximum separation distance is 0 by definition. If $f(\varvec{x})$ is non-convex, then $d_S$ is:

31 $$\begin{aligned} d_S=S(\varvec{x};f^*,\mu )-L_S(\varvec{x};f^*,\mu ) \end{aligned}$$

Because the relative positions of all points are maintained through the $\mu$-subenergy transformation (Corollary 1), the maximum separation distance in the *f*-space ($d_f$) can be expressed based on the reverse transformation of each of the two functions in $d_S$ (Lemma 2):

32 $$\begin{aligned} d_f=&\max _{\varvec{x}\in X_1}\left( -\frac{1}{\mu }\ln (e^{-S(\varvec{x};f^*,\mu )}-1)+f^*- \left( -\frac{1}{\mu }\ln (e^{-L_S(\varvec{x};f^*,\mu )}-1)+f^* \right) \right) \nonumber \\ =&\max _{\varvec{x}\in X_1}\left( \frac{1}{\mu }\ln \left( \frac{e^{-L_S(\varvec{x};f^*,\mu )}-1}{e^{-S(\varvec{x};f^*,\mu )}-1}\right) \right) \end{aligned}$$

By Lemma 3, as $\mu \rightarrow \infty ,L_S\rightarrow S$, therefore:

33 $$\begin{aligned} \lim \limits _{\mu \rightarrow \infty }d_f=\max _{\varvec{x}\in X_1}\left( \lim \limits _{\mu \rightarrow \infty }\frac{1}{\mu }\ln \left( \frac{e^{-L_S(\varvec{x};f^*,\mu )}-1}{e^{-S(\varvec{x};f^*,\mu )}-1}\right) \right) = \max _{\varvec{x}\in X_1}\left( \frac{1}{\infty }\ln (1)\right) =0,\quad \forall \varvec{x}\in X_1 \end{aligned}$$

From the definition of a limit and because $d_f$ is always positive, $\forall \epsilon _f>0$ there exists $c_f:d_f\le \epsilon _f$, $\forall \mu >c_f$. $\square$

Thus, by Theorem 8, an exact $\mu$-subenergy-$\alpha$BB underestimator can produce better lower bounds than the exact $\alpha$BB underestimator of the original function, in an $f_1$-sub-optimal domain. As the value of $\mu$ grows, the maximum separation distance in the *f*-domain will arbitrarily approach zero.

### Properties for $\alpha$ calculated with interval arithmetic

**Fig. 5:**
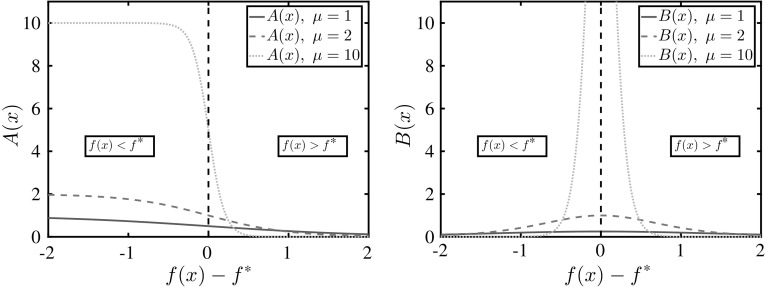
Behaviour of second derivative coefficients $A(x)=\frac{\mu }{1+e^{\mu (f(\varvec{x})-f^*)}}$, $B(x)=\frac{\mu ^2e^{\mu (f(\varvec{x})-f^*)}}{(1+e^{\mu (f(\varvec{x})-f^*)})^2}$ depending on the sign of $f(\varvec{x})-f^*$, for different values of $\mu$

The $\varvec{\alpha }$ vector of an $\alpha$BB relaxation may be calculated in a number of ways. A standard method to produce a rigorous $\varvec{\alpha }$ is interval arithmetic, i.e., to produce an interval Hessian matrix derived from the symbolic expressions of the second derivatives of *f*, and subsequently use an eigenvalue bounding theorem [2, 47] to derive eigenvalue bounds of that matrix. Due to the underlying mathematical structure, and in particular due to the presence of the expression $f(\varvec{x})-f^*$ in the derivatives of the $\mu$-subenergy function, the $\mu$-subenergy method does not seem to benefit from eigenvalue bounds estimated through current interval arithmetic techniques. This is because the flattening effect is dependent on the sign of the expression $f(\varvec{x})-f^*$, as illustrated in Fig. 5. In particular, when $f(\varvec{x})>f^*$, both $A(\varvec{x})$ and $B(\varvec{x})$ vanish. Deriving this information using interval calculations, so that a node *X* may be subenergy-fathomed, is only possible using a result where the interval lower bound of *f* over $X,\underline{f}^{\mathrm {I}}$ is such that $\underline{f}^{\mathrm {I}}>f^*~\forall \varvec{x}\in X$. However, this constitutes a circular requirement, i.e., this node may only be fathomed by making use of information that can be used to fathom it anyway. The proposed method would benefit greatly from rigorous bounds derived through geometrical means; nevertheless, to the authors’ best knowledge, such a technique does not yet exist.

### Properties for $\alpha$ calculated using sampling 

Rigorous $\varvec{\alpha }$ calculations are not always possible or practical [17, 25, 57]. For instance, rigorous $\varvec{\alpha }$ calculations are generally not possible in black-box problems. Despite being primarily employed as a deterministic method, $\alpha$BB has been used successfully in the context of incomplete methods [40] multiple times. Westerberg and Floudas [57] calculated $\varvec{\alpha }$ heuristically, using uniform sampling, in order to calculate all transition states of potential energy surfaces. Esposito and Floudas [17] solved the Respiratory Mechanical Model described in [14] using constant values of $\varvec{\alpha }$, as well as numerically calculated ones, while Klepeis et al. [25] showed that $\alpha$BB can be used to guide a conformational space annealing (CSA) algorithm, producing a powerful hybrid algorithm.

Calculation of $\varvec{\alpha }$ using a rectangular sampling grid of points $\{\varvec{x}_k\}$ is particularly attractive for this method. Sampling eliminates the dependency between flattening and an a priori sign for $f(\varvec{x})-f^*$, therefore allowing the user to exploit the special structure. More importantly however, sampling serves the primary goal of this paper: it allows a computational comparison of the tightest possible underestimators (subject to sampling accuracy) produced using $\mu$-subenergy and classical $\alpha$BB. This is fundamental to this work because it enables us to demonstrate that it is theoretically possible to achieve arbitrarily tight bounds using $\alpha$BB, even in nodes where Theorem 2 is not satisfied. This comparison is otherwise impossible to make unless closed-form eigenvalue limits can be derived, which, according to the Abel Ruffini theorem [13], is impossible for matrices of dimension greater than 4. In the numerical experiments that follow, a rectangular sampling grid is used, which always contains all the vertices of the sampling box.

## Illustrative example

Let us return to the original example in order to demonstrate the proposed underestimation strategy. Consider once again the problem of underestimating $f(x)=\sin (5x)+x^2+2,\,x\in X=[-2,-1]$. Assume that the global minimum $f^\dagger =1.09$ has already been found, and set $f^*=f^\dagger$. Furthermore, let us choose a desired $f_1$-sub-optimal precision $\epsilon =10^{-3}$ and a tolerance $c = 0.69$ such that $f_1=1.78$ is just below the next best local minimum. The resulting value of $\mu$ is $\mu = -\frac{\ln (e^\epsilon -1)}{c}=10.0$.

**Fig. 6:**
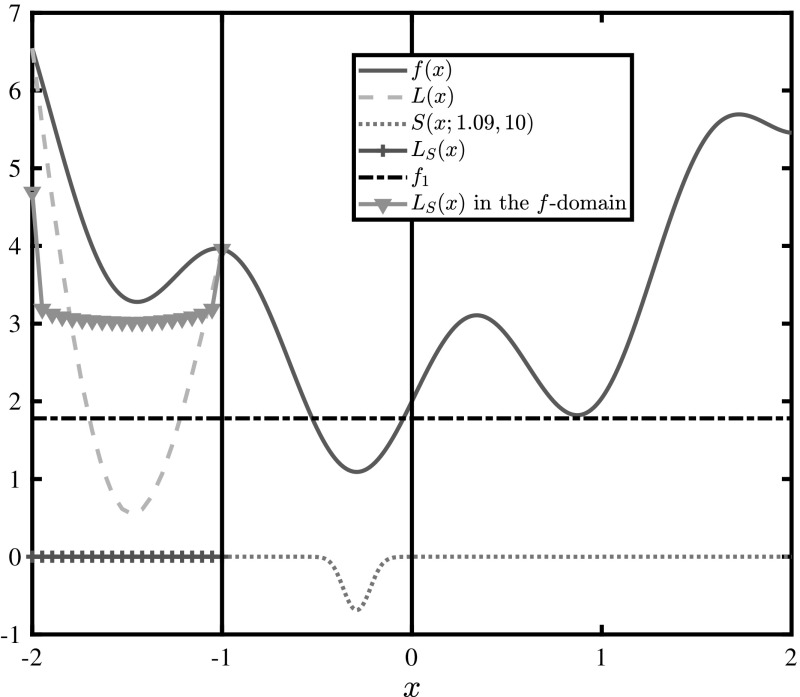
Illustration of the tightest $\alpha$BB underestimators of $f(x)=\sin (5x)+x^2+2$ and its $\mu$-subenergy function, for $x\in [-2,-1]$, $f^*=1.09$, and $\mu =10$

Using this value of $\mu$ guarantees that all points with values greater than 1.78 in the *f*-space will have *S* values between 0 and $-\epsilon$ in the $\mu$-subenergy domain, as illustrated in Fig. 6.

Initially, $\alpha$BB underestimators for both functions are built using an extremely fine resolution of 100,000 points, which yields a very precise value of $\alpha$ for the original problem, i.e., $\alpha =10.98$, while the corresponding value of $\alpha$ for the $\mu$-subenergy problem is $\alpha _S=1.79\cdot 10^{-8}$. The resulting underestimators are displayed in Fig. 6. Even though the tightest possible $\alpha$BB underestimator of *f* does not allow fathoming this particular sub-domain, the $\mu$-subenergy underestimator does.

Lemma 2 is used to map the $\mu$-subenergy underestimator back to the *f*-space for a better overview of the underestimator, and also to map the $\mu$-subenergy lower bounds back to the *f*-space, for easier comparison. It is observed in Fig. 6 that the exact $\mu$-subenergy underestimator is much tighter than the exact classical $\alpha$BB underestimator; enough so that the node may now be fathomed.

Similar results may be achieved using much lower sampling resolutions. The lower bounds on *f* that are achieved using different sampling resolutions for node *X* are displayed in Table 1. All *f*-lower bounds using the $\mu$-subenergy function are consistently tighter than the $\alpha$BB *f*-lower bounds on the original function, with their accuracy improving, as expected, with more sampling points. For this simple example, one sampling point (excluding the vertices of the node) appears to be sufficient to fathom this $f_1$-sub-optimal node. In fact, because the value of $f^* =1.09$ is considerably smaller than $f^m=3.28$, any sample will be adequate to produce a fathoming underestimator.

**Table 1:** Lower bounds on $f(x)=\sin (5x)+x^2+2$, $x\in [-2,-1]$ using $\mu =10,~f^*=1.09$ (sampling density given as the number of samples per unit length)

# sampling points (excluding vertices)	Sampling density	*f*-lower bound using $\alpha$BB	*f*-lower bound using subenergy-$\alpha$BB
1	3	0.54	3.30
3	5	0.54	3.14
5	7	0.54	3.04
7	9	0.54	3.11
10	12	0.54	3.01
50	52	0.54	3.01
100	102	0.54	3.00
1000	1002	0.54	3.00
10,000	10,002	0.54	3.00
100,000	100,002	0.54	3.00

For lower sampling resolutions (i.e., a density of 3 points per unit length) the lower bound is less accurate,^2^ meaning that a false positive is possible, i.e., fathoming the node which holds the global solution due to poor sampling. This is further investigated by performing tests in a node which contains the global solution ($x\in [-1,0]$). The sampling results are shown in Table 2, where robustness with respect to the sampling size is observed, and even for very small sample sizes the node is not incorrectly fathomed.

**Table 2:** Lower bounds on $f(x)=\sin (5x)+x^2+2$, $x\in [-1,0]$ using $\mu =10,~f^*=1.09$

# sampling points (excluding vertices)	Sampling density	*f*-lower bound using $\alpha$BB	*f*-lower bound using subenergy-$\alpha$BB
1	3	$-$ 1.39	0.95
3	5	$-$ 1.39	0.95
5	7	$-$ 1.39	0.16
7	9	$-$ 1.39	0.58
10	12	$-$ 1.47	0.23
50	52	$-$ 1.51	0.09
100	102	$-$ 1.51	0.10
1000	1002	$-$ 1.51	0.09
10,000	10,002	$-$ 1.51	0.09
100,000	100,002	$-$ 1.51	0.09

It is possible however to incorrectly fathom the node if all sampling points happen to be in flattened parts of the domain. In order to demonstrate this, we widen the bounds to $x\in [-1,2]$. The resulting lower bounds are presented in Table 3, where for sampling sizes smaller than 10, the method is not consistently reliable.

**Table 3:** Lower bounds on $f(x)=\sin (5x)+x^2+2$, $x\in [-1,2]$ using $\mu =10,~f^*=1.09$

# sampling points (excluding vertices)	Sampling density	*f*-lower bound using $\alpha$BB	*f*-lower bound using subenergy-$\alpha$BB
1	1	$-$ 22.16	**2**.**14**
3	1.66	$-$ 22.16	**2**.**14**
5	2.33	$-$ 22.16	0.12
7	3	$-$ 22.28	**1**.**53**
10	4	$-$ 22.45	$-$ 6.64
50	17.33	$-$ 23.30	$-$ 7.85
100	34	$-$ 23.30	$-$ 7.73
1000	334	$-$ 23.30	$-$ 7.84
10,000	3334	$-$ 23.30	$-$ 7.85
100,000	33,334	$-$ 23.30	$-$ 7.85

The node is incorrectly fathomed for small sampling sizes (cells marked in bold)

Overall, it is observed that, in this example, lower bounds on *f* are consistently tighter than standard $\alpha$BB when $\mu$-subenergy underestimation is used.

## Computational experiments

In this section we present results using the sampling approach with $\mu$-subenergy underestimators on a selection of widely-used optimization test functions. The purpose of these tests is to validate our theoretical results and demonstrate that, despite being a proof-of-concept methodology, $\mu$-subenergy underestimation may be a viable alternative to classical $\alpha$BB in a heuristic context. The construction of all underestimators and the calculation of their bounds are performed in MATLAB R2016a using INTLAB V6 [44], and the code is publicly available at https://zenodo.org/deposit/238684.

The tests are performed on a set of 16 test functions [1, 22, 41–43] in two configurations: (1) in a sub-optimal box and (2) in an optimal box. In order to keep the function ranges in comparable levels, boxes of different sizes are selected, which can be seen in “Appendix”.

In all calculations in this section, $\mu$ is set to $\mu =10$, and $f^*$ for each case study is set to the global minimum of the corresponding function, and all numbers are rounded down to the second decimal. Tables 4 and 5 illustrate the *f*-lower bounds using $\mu$-subenergy and classical $\alpha$BB respectively, in boxes which do not contain the global solution, for different sampling resolutions. In those tables we also provide the approximate lower bound (rounded down to the second digit) of *f* in the corresponding sub-optimal node.

The lower bounds derived using the $\mu$-subenergy method are much tighter than the corresponding ones using standard $\alpha$BB. With the classical $\alpha$BB it is possible to fathom 8 out of 16 nodes. With the $\mu$-subenergy it is possible to fathom 6 more nodes, otherwise impossible to fathom using the $\alpha$BB relaxation of the original function. Interestingly, the sub-optimal nodes of problems hatflda (fathomed by $\alpha$BB) and biggs5 (not fathomed by $\alpha$BB) are not fathomed. We observe that the bounds are not invalid, as they are smaller than the values at the global minimum, which indicates that the value of $\mu$ may not be large enough. In the same table we also present the corresponding calculations using $\mu =20$ for these two functions, where the larger value of the subenergy magnitude enables us to fathom both nodes.

**Table 4:** Lower bounds on each function using $\mu$-subenergy with sampling and $\mu =10$ (boxes do not contain the global minimum)

Function	Dimensions	Global solution	Approximate LB	Sampling points					
				10	50	100	1000	2500	5000
rastrigin	2	0.00	2.00	*3*.*60*	*3*.*54*	*3*.*47*	1.43	1.38	1.38
rosenbrock	2	0.00	1.32	0.87	0.87	0.87	0.87	0.87	0.87
ackley	2	0.00	3.62	3.54	3.54	3.40	3.17	3.17	3.16
beale	2	0.00	14.20	3.60	3.60	3.60	3.60	3.60	3.60
goldstein	2	3.00	7.46	6.54	6.47	6.49	6.47	6.47	6.47
box3	3	0.00	3.06	0.94	0.94	0.94	0.94	0.94	0.94
allinit	3	16.71	23.16	20.24	20.24	20.24	20.24	20.24	20.24
biggs3	3	0.00	3.10	2.58	2.58	2.58	2.58	2.58	2.58
denschnd	3	0.00	5.00	3.54	3.54	3.54	3.54	3.54	3.54
allinitu	4	5.74	14.89	9.35	9.35	9.35	9.35	9.35	9.35
hatflda	4	0.00	0.378	$-$ 0.17	$-$ 0.17	$-$ 0.17	$-$ 0.21	$-$ 0.22	$-$ 0.22
hatflda ($\mu =20$)	4	0.00	0.378	0.094	0.094	0.090	0.066	0.065	0.066
biggs5	5	0.00	0.43	$-$ 0.01	$-$ 0.12	$-$ 0.12	$-$ 0.17	$-$ 0.13	$-$ 0.15
biggs5 ($\mu =20$)	5	0.00	0.43	0.33	0.19	0.19	0.14	0.14	0.14
genhumps	5	0.00	0.72	0.69	0.47	0.47	0.33	0.38	0.33
hs045	5	1.00	1.60	1.34	1.34	1.34	1.34	1.34	1.34
hart6	6	$-$ 3.32	$-$ 3.42E$-$05	$-$ 0.01	$-$ 0.01	$-$ 0.01	$-$ 0.01	$-$ 0.01	$-$ 0.01
s273	6	0.00	1.72	3.60	3.60	0.56	3.60	3.60	0.56

Cells in italics correspond to cases where the lower bounds using $\mu$-subenergy are not valid

**Table 5:** Lower bounds on each function using $\alpha$BB with sampling (boxes do not contain the global minimum)

Function	Dimensions	Global solution	Approx. LB	Sampling points					
				10	50	100	1000	2500	5000
rastrigin	2	0.00	2.00	$-$ 14.60	$-$ 45.07	$-$ 48.57	$-$ 53.69	$-$ 53.98	$-$ 54.17
rosenbrock	2	0.00	1.32	1.33	1.33	1.33	1.33	1.33	1.33
ackley	2	0.00	3.62	1.85	2.22	1.85	1.85	1.84	1.84
beale	2	0.00	14.20	$-$ 15.24	$-$ 15.24	$-$ 15.24	$-$ 15.24	$-$ 15.24	$-$ 15.24
goldstein	2	3.00	7.46	1.6	1.36	1.35	1.35	1.35	1.35
box3	3	0.00	3.06	$-$ 1.60E+06	$-$ 1.60E+06	$-$ 1.60E+06	$-$ 1.60E+06	$-$ 1.60E+06	$-$ 1.60E+06
allinit	3	16.71	23.16	20.66	20.66	20.66	20.66	20.66	20.66
biggs3	3	0.00	3.10	3.10	3.10	3.10	3.10	3.10	3.10
denschnd	3	0.00	5.00	$-$ 23.80	$-$ 23.10	$-$ 23.80	$-$ 23.80	$-$ 23.87	$-$ 23.86
allinitu	4	5.74	14.89	$-$ 39.15	$-$ 39.88	$-$ 40.02	$-$ 40.02	$-$ 40.00	$-$ 40.03
hatflda	4	0.00	0.37	0.38	0.38	0.38	0.38	0.38	0.38
biggs5	5	0.00	0.43	$-$ 2.42	$-$ 2.42	$-$ 2.42	$-$ 2.42	$-$ 2.42	$-$ 2.42
genhumps	5	0.00	0.72	$-$ 6.63	$-$ 6.63	$-$ 6.63	$-$ 6.63	$-$ 6.63	$-$ 6.63
hs045	5	1.00	1.60	1.60	1.60	1.60	1.60	1.60	1.60
hart6	6	$-$ 3.32	$-$ 3.42E$-$ 05	$-$ 0.01	$-$ 0.01	$-$ 0.01	$-$ 0.01	$-$ 0.01	$-$ 0.01
s273	6	0.00	1.72	1.73	1.73	1.73	1.73	1.73	1.73

**Table 6:** Lower bounds on each function in boxes containing the global minimum using $\mu$-subenergy with sampling and $\mu =10$

Function	Dimensions	Global solution	Sampling points					
			10	50	100	1000	2500	5000
rastrigin	2	0.00	*3.60*	*3.43*	*3.60*	$-$ Inf	$-$ Inf	$-$ Inf
rosenbrock	2	0.00	0.00	$-$ 24.73	$-$ 66.09	$-$ Inf	$-$ Inf	$-$ Inf
ackley	2	0.00	0.00	0.00	$-$ 0.06	$-$ 25.29	$-$ 23.13	$-$ 26.14
beale	2	0.00	$-$ 7.98	$-$ 18.18	$-$ 15.91	$-$ 18.47	$-$ 18.97	$-$ 18.89
goldstein	2	3.00	3.00	3.00	3.00	$-$ 65	$-$ Inf	$-$ Inf
box3	3	0.00	$-$ 1.24E$-$ 04	$-$ 3.39	$-$ Inf	$-$ Inf	$-$ Inf	$-$ Inf
allinit	3	16.71	*17.75*	*16.07*	13.27	1.96	2.31	$-$ 1.49
biggs3	3	0.00	$-$ 0.52	$-$ 0.61	$-$ 0.82	$-$ 1.43	$-$ 1.48	$-$ 1.49
denschnd	3	0.00	$-$ 0.11	$-$ 2.83	$-$ 4.30	$-$ 11.62	$-$ 11.45	$-$ 12.52
allinitu	4	5.74	5.74	4.12	4.74	4.34	3.24	3.24
hatflda	4	0.00	$-$ 0.25	$-$ 0.50	$-$ 0.72	$-$ 0.76	$-$ 0.78	$-$ 0.77
biggs5	5	0.00	$-$ 1.00	$-$ 5.25	$-$ 5.25	$-$ 6.28	$-$ 7.08	$-$ 8.71
genhumps	5	0.00	0.00	$-$ 0.21	$-$ 0.21	$-$ 0.42	$-$ 0.48	$-$ 4.27
hs045	5	1.00	$-$ 3.60	$-$ 3.60	$-$ 3.60	$-$ 3.60	$-$ 3.60	$-$ 3.60
hart6	6	$-$ 3.32	$-$ 0.73	$-$ 0.73	$-$ 3.16	$-$ 3.80	$-$ 3.80	$-$ 5.27
s273	6	0.00	0.00	0.00	0.00	0.00	0.00	0.00

Cells in italics correspond to cases where the lower bounds using $\mu$-subenergy are not valid

**Table 7:** Lower bounds on each function in boxes containing the global minimum using $\alpha$BB with sampling

Function	Dimensions	Global solution	Sampling points					
			10	50	100	1000	2500	5000
rastrigin	2	0.00	0.00	$-$ 755.86	$-$ 408.15	$-$ 838.82	$-$ 841.55	$-$ 843.37
rosenbrock	2	0.00	$-$ 92.09	$-$ 92.09	$-$ 92.09	$-$ 92.09	$-$ 92.09	$-$ 92.09
ackley	2	0.00	$-$ 20.15	$-$ 19.39	$-$ 21.16	$-$ 22.38	$-$ 22.53	$-$ 22.60
beale	2	0.00	$-$ 6.82	$-$ 8.07	$-$ 8.43	$-$ 8.44	$-$ 8.46	$-$ 8.45
goldstein	2	3.00	$-$ 6.66E+04	$-$ 6.66E+04	$-$ 6.66E+04	$-$ 6.66E+04	$-$ 6.67E+04	$-$ 6.67E+04
box3	3	0.00	$-$ 1.95E+07	$-$ 1.95E+07	$-$ 1.95E+07	$-$ 1.95E+07	$-$ 1.95E+07	$-$ 1.95E+07
allinit	3	16.71	$-$ 93.49	$-$ 93.49	$-$ 93.49	$-$ 93.49	$-$ 93.49	$-$ 93.49
biggs3	3	0.00	$-$ 0.02	$-$ 0.02	$-$ 0.02	$-$ 0.02	$-$ 0.02	$-$ 0.02
denschnd	3	0.00	$-$ 45.99	$-$ 45.99	$-$ 45.99	$-$ 49.12	$-$ 49.98	$-$ 50.01
allinitu	4	5.74	4.03	3.93	3.94	3.93	3.93	3.93
hatflda	4	0.00	$-$ 0.12	$-$ 0.12	$-$ 0.12	$-$ 0.12	$-$ 0.12	$-$ 0.12
biggs5	5	0.00	$-$ 17.05	$-$ 17.05	$-$ 17.05	$-$ 17.05	$-$ 17.05	$-$ 17.05
genhumps	5	0.00	$-$ 30.10	$-$ 30.10	$-$ 30.10	$-$ 30.10	$-$ 30.10	$-$ 32.13
hs045	5	1.00	$-$ 0.72	$-$ 0.72	$-$ 0.72	$-$ 0.72	$-$ 0.72	$-$ 0.72
hart6	6	$-$ 3.32	$-$ 10.14	$-$ 10.14	$-$ 21.88	$-$ 33.89	$-$ 33.89	$-$ 34.14
s273	6	0.00	0.00	0.00	0.00	0.00	0.00	0.00

No nodes are incorrectly fathomed

The next step is to investigate the sampling size necessary in order to avoid false positives. Thus, the $\mu$-subenergy method is used to build relaxations in boxes which contain the global minimum. The results for $\mu$-subenergy and $\alpha$BB respectively are presented in Tables 6 and 7, where it may be observed (Table 6) that we avoid false positives in every case, with the exception of the Rastrigin function, where a sampling size greater than 100 samples is necessary. The data for large sample sizes may lead to Hessian matrices that contain $-\infty$. This occurs because, in the process of flattening sub-optimal domains, the $\mu$-subenergy function inevitably increases the curvature in optimal domains. The sharp bend of the $\mu$-subenergy function as it transitions from a flat region to an optimal one may produce eigenvalues of very high magnitude in $X_1\cap X_0$. However, this does not pose a problem in a branch-and-bound context because such behaviour flags a region as optimal: these infinitely negative eigenvalues yield a LB of minus infinity, which correctly preserves the node for further branching. The corresponding results for $\alpha$BB suggest that classical $\alpha$BB is more robust in all tests in optimal nodes, as no node is incorrectly fathomed, and fewer samples are required to achieve reliable lower bounds. Although sampling is generally sensitive to the dimensionality of the problem, we find that 100 samples are sufficient in most cases investigated, with up to 1000 samples being necessary to properly capture the curvature of the functions. In this small test set, increasing the number of dimensions does not appear to require significantly more samples to achieve similar levels of accuracy.

Collectively, the tests confirm our theoretical expectation: if exact eigenvalues are available (indicated by the results for high sampling resolutions across all tests), the $\mu$-subenergy methodology may produce much tighter bounds than classical $\alpha$BB in the domains were these bounds may actually be used for fathoming, i.e., $f_1$-sub-optimal domains.

## Conclusions

In this paper we proved that a general $\alpha$BB underestimator may only give an exact lower bound if certain conditions are satisfied (Theorems 1 and 2). We then presented a new methodology to produce $\alpha$BB underestimators which allow the fathoming of nodes even in cases where the rigorous conditions described in Theorems 1 and 2 are not satisfied.

This was achieved by introducing a modified transformation, the $\mu$-subenergy function, based on the concept of subenergy functions initially proposed for tunneling [8, 12], and by deriving a number of useful properties. Given two arbitrary pre-defined tolerances in the *f*-space and *S*-space, a parameter $\mu$ may be calculated such that all values greater than the *f*-tolerance (*c*) are mapped between 0 and the *S*-tolerance $(-\epsilon )$. Thus, it is possible to bound values in certain sub-domains of $X_0$ between two barriers in *S*-space, by controlling the mapping. It was shown that these bounding properties extend to the derivatives of the $\mu$-subenergy function, where we provided the conditions to calculate $\mu$ in order to impose arbitrary bounds. Using this methodology, domains otherwise impossible to fathom using a standard $\alpha$BB relaxation may theoretically be fathomed.

This method was shown not to benefit from the established method of deriving a rigorous $\varvec{\alpha }$ vector using interval arithmetic [2]; thus, there is no method currently known to the authors which may be employed both to calculate the $\varvec{\alpha }$ vector rigorously, and to produce tighter bounds than classical $\alpha$BB; we consider this to be a promising subject of future research.

The method is not beyond practical use however; a series of numerical tests were performed in order to compare the tightest possible $\mu$-subenergy underestimator against the tightest possible $\alpha$BB underestimator using sampling-based eigenvalues. The $\mu$-subenergy method was shown in all tests to be able to fathom domains which may not theoretically be fathomed using classical $\alpha$BB, as predicted by the theory. Thus, the $\mu$-subenergy theory, beyond its purpose as a proof-of-concept that the $\alpha$BB theoretical underestimation limit might be overcome, may be applied in cases where $\alpha$BB is used as a heuristic method.

This method successfully demonstrates that, through appropriate manipulation, an $\alpha$BB underestimator may be used to produce tighter bounds than the theoretical conditions derived in this paper would allow, up to a maximum separation distance arbitrarily close to zero. The $\mu$-subenergy methodology is a promising starting point for future research regarding methods to overcome the theoretical limits of superposition-type underestimators.

**Data statement**

Data underlying this article can be accessed at zenodo.org at https://zenodo.org/deposit/238684, and used under the Creative Commons Attribution licence.

## Appendix

See Table 8.

**Table 8:** Nodes used in numerical experiments

Function	Optimal node	Sub-optimal node
rastrigin	[$-$1 2 $-$2 1]	[$-$2 $-$1 $-$2 $-$1]
rosenbrock	[0.5 1.5 0.5 1.5]	[0.15 .25 0.15 .25]
ackley	[$-$1 1 $-$1 1]	[$-$2 $-$1 $-$2 $-$1]
beale	[2 4 0 1]	[1 2 1 2]
goldstein	[$-$1 1 $-$1.5 $-$0.5]	[0.01 0.3 $-$0.9 $-$0.8]
box3	[$-$9 $-$8 $-$9 $-$8 $-$1 1]	[$-$9 $-$8.5 $-$9 $-$8.5 1 1.5]
allinit	[$-$2 0 1 2 $-$2 $-$1]	[$-$2 $-$1.5 1.5 2 $-$1 0]
biggs3	[0.5 1.5 9 11 4 6]	[2 3 1 2 1 2]
denschnd	[$-$1 1 $-$1 1 $-$1 1]	[1 2 1 2 1 2]
allinitu	[1 2 $-$0.5 0.5 $-$0.5 0.5 $-$1 0]	[0 1 1 2 1 2 1 2]
brownden	[$-$11.6 $-$11.4 13 13.5 $-$0.5 $-$0.3 0 .3]	[$-$1.2 $-$1 1 1.2 1 1.2 1 1.2]
hatflda	[0.5 1.5 0.5 1.5 0.5 1.5 0.5 1.5]	[1.5 2.5 1.5 2.5 1.5 2.5 1.5 2.5]
biggs5	[9 11 0 2 $-$6 $-$4 $-$2 0 4 5]	[1 2 2 3 $-$3 $-$2 $-$3 $-$2 1 2]
genhumps	[$-$1 1 $-$1 1 $-$1 1 $-$1 1 $-$1 1]	[1 2 1 2 1 2 1 2 1 2]
hs045	[0.5 1.5 1.5 2.5 2.5 3.5 3.5 4.5 4.5 5.5]	[2 3 1 2 1 2 1 2 1 2]
hart6	[0 1 0 1 0 1 0 1 0 1 0 1]	[1 2 1 2 1 2 1 2 1 2 1 2]
s273	[0.5 1.5 0.5 1.5 0.5 1.5	[$-$0.1 0.9 0.5 1.5 0.5 1.5
	0.5 1.5 0.5 1.5 0.5 1.5]	0.5 1.5 0.5 1.5 0.5 1.5]

The variable bounds are presented in the following format: $x_1^L~x_1^U~x_2^L~x_2^U\dots ~x_N^L~x_N^U$

## Footnotes

1 This *a* [only used in Eq. (13)] should not be confused with the $\alpha$ symbol used for $\alpha$BB.

2 In the first row of Table 1, $\underline{f}_S>f^m$.
